# Interpreting the prevalence of musculoskeletal pain impacting Italian and Peruvian dentists likewise: A cross-sectional study

**DOI:** 10.3389/fpubh.2023.1090683

**Published:** 2023-02-09

**Authors:** Monica Macrì, Natali V. Galindo Flores, Riccardo Stefanelli, Francesco Pegreffi, Felice Festa

**Affiliations:** ^1^Department of Innovative Technologies in Medicine and Dentistry, University “G. D'Annunzio” of Chieti-Pescara, Chieti, Italy; ^2^Unimedical Group, Bologna, Italy; ^3^University of Bologna, Bologna, Italy; ^4^Department for Life Quality Studies, University of Bologna, Bologna, Italy; ^5^Department of Biomolecular Sciences, University of Urbino Carlo Bo, Urbino, Italy

**Keywords:** musculoskeletal pain, work-related disorders, drug assumption, dental practitioners, dentists

## Abstract

**Background:**

Musculoskeletal pain is a frequent condition among dental practitioners due to working in prolonged static isometric/eccentric contraction. The study aimed to describe musculoskeletal pain prevalence and the interplay between environmental conditions, lifestyle, and drugs consumed among Italian and Peruvian dentists.

**Methods:**

A 18 multiple choice questionnaire was administered to Peruvian and Italian dental care practitioners. A total of 187 questionnaires were submitted. One hundred sixty-seven questionnaires were selected for the analysis, including 86 questionnaires from Italy, and 81 from Perú. The study examined musculoskeletal pain presence in dental practitioners. The prevalence of musculoskeletal pain presence was analyzed considering different parameters: gender, age, type of dental practitioners, specialization in dentistry, hours of work per day, years of work, physical activity, localization of musculoskeletal pain and the influence of musculoskeletal pain on the performance on work.

**Results:**

The selected questionnaires for analysis were 167 (67 from Italy and 81 from Perú). Male and female participants were numerically equal. Most of the dental practitioners were dentists. The percentage of dentists who present musculoskeletal pain is 87.2% in Italy and 91.4% in Perú (*p* < 0.05).

**Conclusion:**

Musculoskeletal pain represents a very diffused condition in dental practitioners. The results about the prevalence of musculoskeletal pain show how the two populations (Italian and Peruvian) are very similar despite the geographical distance. Nevertheless, the high percentages of musculoskeletal pain in dental practitioners translate into the need to use solutions to reduce its onsets, such as improving ergonomics and physical activity.

## Introduction

Work-related musculoskeletal disorders are a frequent and primary burden among dental care practitioners, with symptoms often starting early and progressively worsening over time (1).

Work-related musculoskeletal disorders associated with exposure to risk factors in the workplace (1) are the second most common work-related problem worldwide (2). A link of paramount importance that should be considered is the theoretical model, which clearly explains the relationship with the development of industrial science and technology, changes in production practices, forced posture movements of low-load, rapid rhythms and high repetition for a long duration, all leading to musculoskeletal disorders. These work practices can easily lead to local muscle fatigue and, in severe cases, local musculoskeletal disease.

Lee et al. (1) reported that years of work and perceived health status increased the prevalence of shoulder symptoms.

Leggat et al. (3) identified a dose-response relationship between specific shoulder diseases and hands at/above shoulder level, repetitive movements, forceful work, and hand/arm vibrations. The situation mentioned above is progressively generated by forced activity maintaining muscular imbalance and asymmetrical positions for a long time.

This item is gradually acquiring more interest, and the scientific community is starting to promote studies to delineate biomechanical factors' role better.

In particular, neck inclination/rotation, forward bending with loss of cervical and lumbar lordosis, and raised arms working in prolonged static isometric/eccentric contraction represent the main risk factors for musculoskeletal pain (2, 4).

Furthermore, pain should be interpreted as the first adaptive biological “alarm bell” revealing musculoskeletal overload. Thus, if the muscle over-work is not promptly controlled and interrupted, it could become a musculoskeletal problem and progressively evolve into a tangible disease (5).

These musculoskeletal issues, such as muscles, tendons, joints, cartilage, nerves, ligaments and vertebral column burdens, are related to unsuitable body positions and forced postures (6).

Over time, the progressive biomechanical overload determined by counteracting continual dental procedure repetition, often in a forceful manner and with a lack of recovery time, repeatedly leads to pain, spasms, joint rigidity, shivers, and disturbances in daily life.

Several postural factors were found to be associated with musculoskeletal symptoms in the shoulder. “Standing for a long time” and “sitting for a long time” were associated with an increased prevalence of upper harm symptoms.

Applying precise rules within the ergonomics guidelines is essential to avoid the onset of disease in dental professionals and, consequently, the development of tiredness, tingling, pain, and numbness in the shoulders, lower back, and neck (7–9).

The literature reports that dental professionals worldwide complain of musculoskeletal pain at least once in a lifetime, ranging from 64 to 93% (10).

Even when the ergonomic working precautions are respected, dental professionals are subjected to mental and physical stress and forced daily to control pain using indiscriminately under-the-counter drugs, such as painkillers (11, 12).

Due to multi-factorial settings and environmental conditions, the aforementioned complex scenario has never been taken into account previously, and drug assumptions were underestimated.

### Aim of the study

The study aimed to describe musculoskeletal pain prevalence and the interplay between environmental conditions, lifestyle, and drugs consumed among Italian and Peruvian dentists.

## Materials and methods

Between March 2020 and March 2021, a cross-sectional observational study was conducted using a questionnaire to detect a snapshot of selected characteristics among Italian and Peruvian dentistry communities.

A propaedeutic phase consisted of formulating the questionnaire items, selecting subjects and specific areas to investigate.

The study investigated different interest areas using 18 multiple-choice questions administered as a survey in Italian and Spanish to reach out to Italian and Peruvian dentists (Supplementary Table 1). All the questions were discussed by the workforce group, given that a specific questionnaire for dentists is unavailable. That's why the questionnaire employed was not validated.

Two operators blindly delivered the questionnaires, through a validated virtual platform (Survey Monkey, https://it.surveymonkey.com), through mailing lists, wherein the Italian and Peruvian participants found all the instructions to fill in the questionnaire quickly.

Thus, the working group could reach out to many dentists in Italy and Perú. The IP code was registered and considered a reliable identification parameter to identify each participant individually.

The workforce did not calculate the sample size because we employed survey methodology. The respondents were chosen using dedicated dentists' mailing lists and social network pages. The choice was made to reach out to people suitable to include in our study.

To avoid misunderstandings during questionnaire compilation and increase compliance/adherence, participants were (R1-2) approached and encouraged to complete it and carefully read the explanation text before the questions. Incomplete questionnaires were excluded.

*Inclusion criteria* were: a stable occupation in the dental profession, a degree in dentistry or dental hygiene, and an age between 20 and 70 years.

*Exclusion criteria* were: retired professionals, undergraduate students, significant accidents, traumas or medical conditions that lead to permanent musculoskeletal disabilities.

The approval and ethical review of the procedures and methodologies for collecting data for survey-based research were provided by the University Gabriele D'Annunzio Ethical Board.

### Statistical analysis

An Excel document was used to collect the data. Each row corresponded to a responder, while each question corresponded to a column. The possible answers for each question, where it is possible, were identified as Boolean variables (for example, yes = 1, no = 0). R-studio software was used for statistical analysis, and partially incomplete questionnaires were counted as NA answers. The Chi-square test was used to examine the differences between variables; even if the set was small, we used the test to investigate independence. The *p*-value was set to 0.05.The following parameters were considered, namely gender, age, occupation, height, dental occupation, working hours per day, years of work, physical activity, physical activity per week and presence of WMSD disease. (R2) To ensure that the population was representative, we identified a margin of error of 5%, and to have a good confidence level, we stayed within 90%. To obtain these results, we identified a sample population of 200 people. As expected, we then noticed a non-response rate of 10% for the Italian population and 13% for the Peruvian population. The goal was to get 100 people of both nationalities, and by doing the ratio of the expected people to the probable non-response rate of about 10%, we sent the questionnaire to a total of ~1,000 people for both groups. In conclusion, we also had to exclude a priori those who had had an upper limb or cervical spine surgery. Instead, we did not exclude people with prior non-serious conditions so that the sample would not be non-significant and because we are not sure if there are direct causes associated with it. For this reason, the questions do not report the previously analyzed matter to create a consonant subgroup analysis.

## Results

### Population socio-demographic description

A total of 187 questionnaires were submitted. Out of these, eight were incorrectly filled in, and 12 were given back incomplete. One hundred sixty-seven questionnaires were selected for the analysis; 86 were submitted from Italy and 81 from Perú. Each group was homogeneous concerning gender.

**Figure 1:**
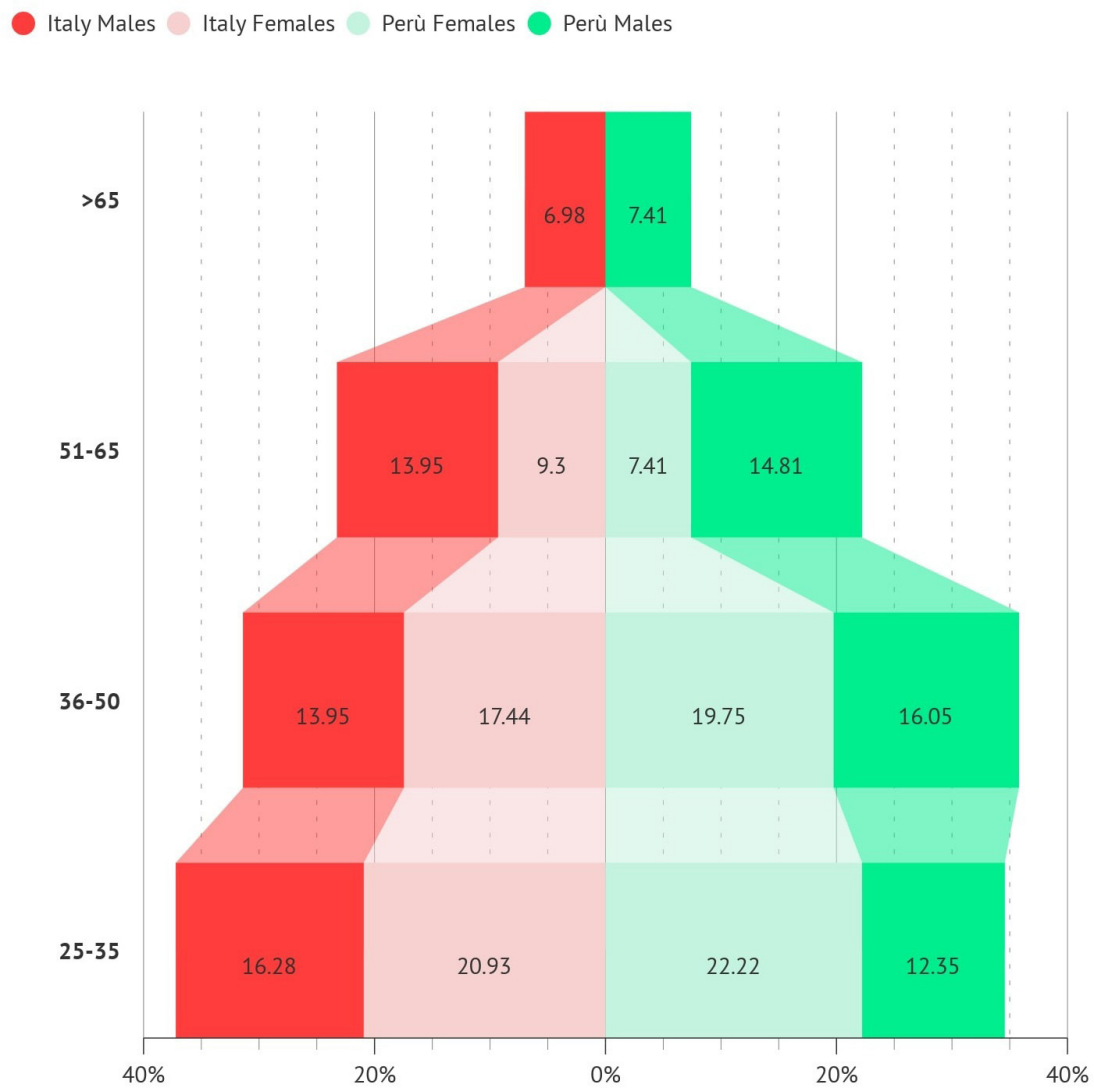
Age range of participants from Peru and Italy.

The age range showed a prevalence of young dental professionals, with ages < 35 years (Figure 1).

Most of the surveyed operators were dentists.

We did not exclude dental hygienists; however, a low emphasis was given due to low power analysis. Operators working in only one discipline were the least represented. Dental specialists' sub-groups included general dentists/endodontists/restorative/periodontists (41% in Italy, 24% in Perú), prosthodontists/implantologists (21% in Italy, 10% in Perú), oral surgeons (17% in Italy, 5% in Perú), orthodontists (7% in Italy and 2% in Perú). Only in Italy, 3% were pediatric orthodontists. Thirty-five percent of Perú answered, “I do not know.”

**Figure 2:**
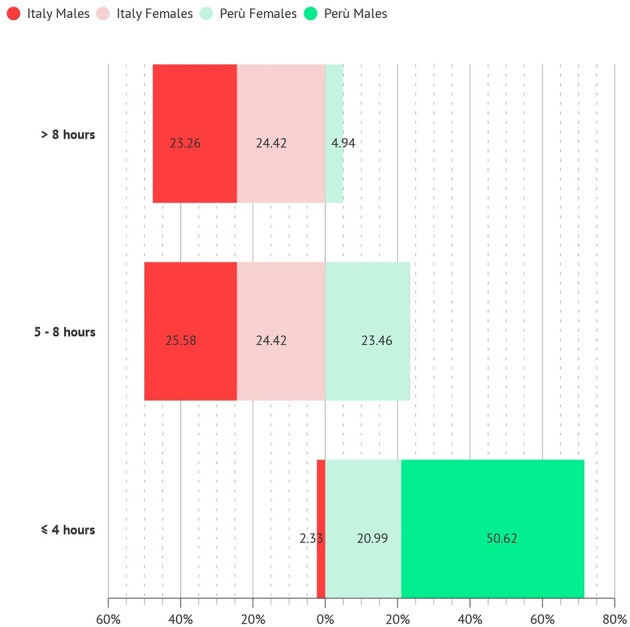
Hours of work per day.

Most of the surveyed personnel worked 5–8 h per day (~50%) or more than 8 h per day (~50%), with no significant difference between Italy and Perú (Figure 2).

**Figure 3:**
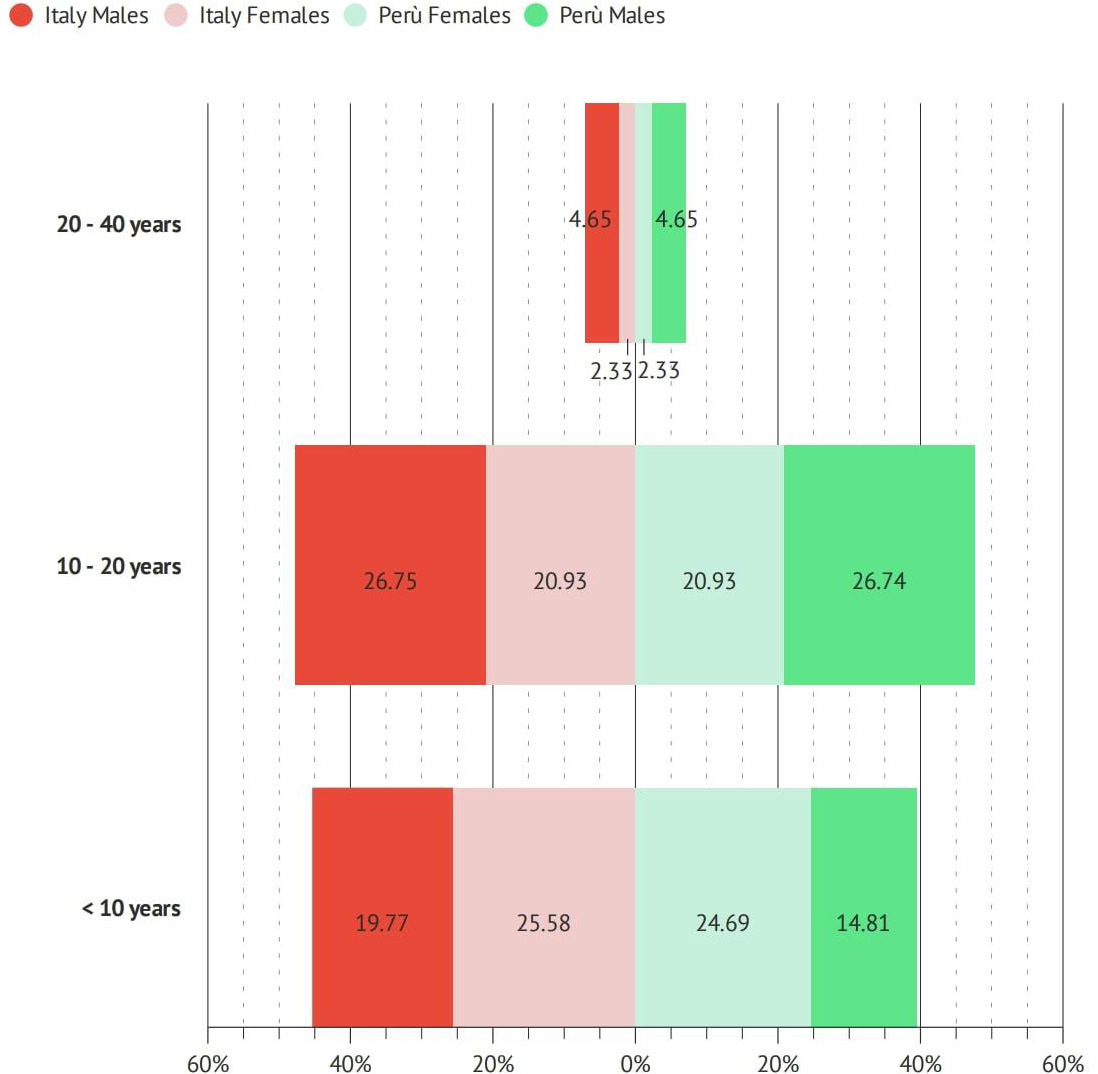
Total years of work of participants.

The number of years of work is uniformly distributed among dental professionals, with no differences between Italy and Perú (Figure 3).

**Figure 4:**
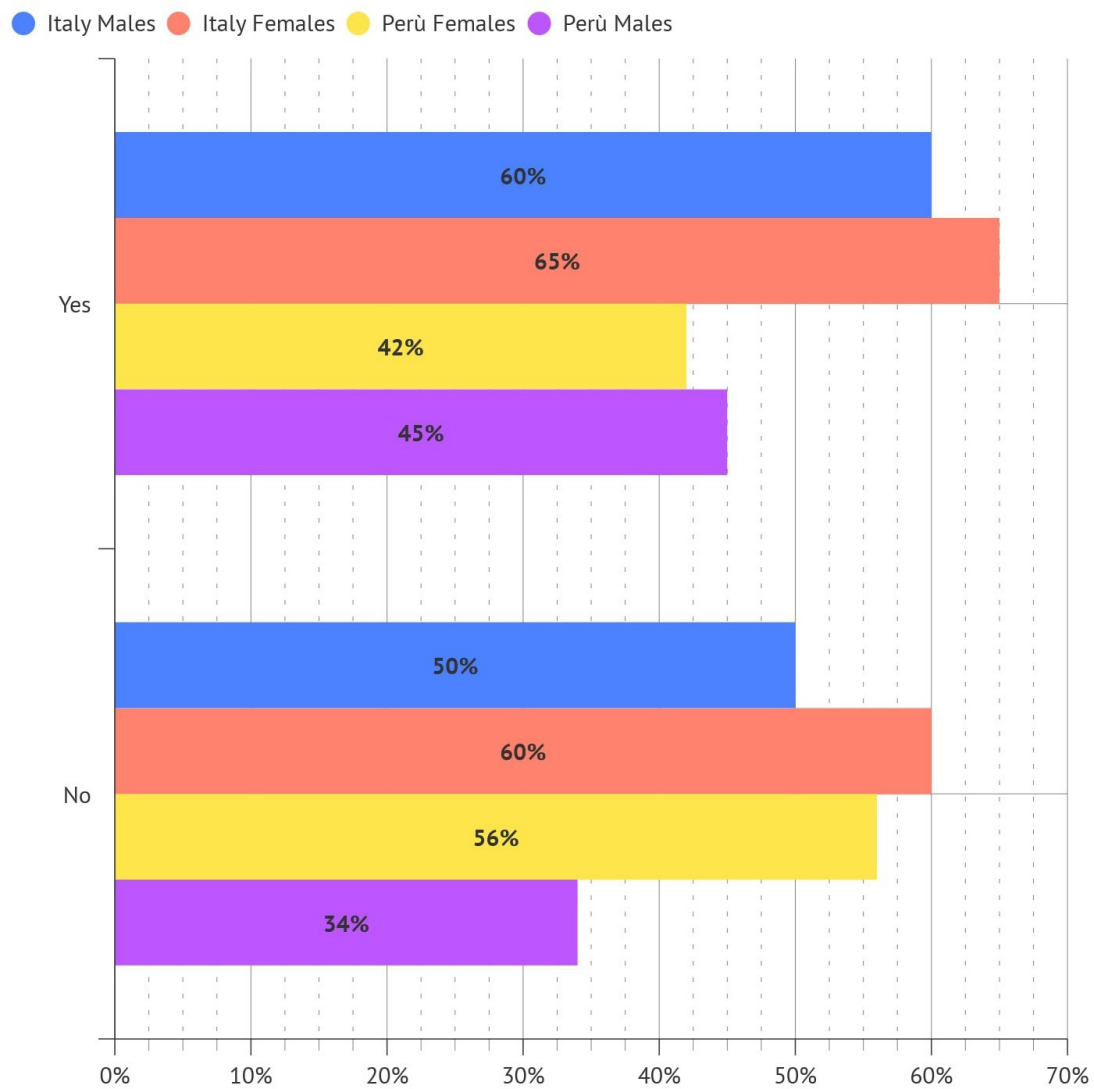
Percentage of dentistry professionals engaging in physical activity.

Among the population, 85% of Italians and 72% of Peruvian have an active lifestyle performing weekly physical activity (Figure 4).

**Figure 5:**
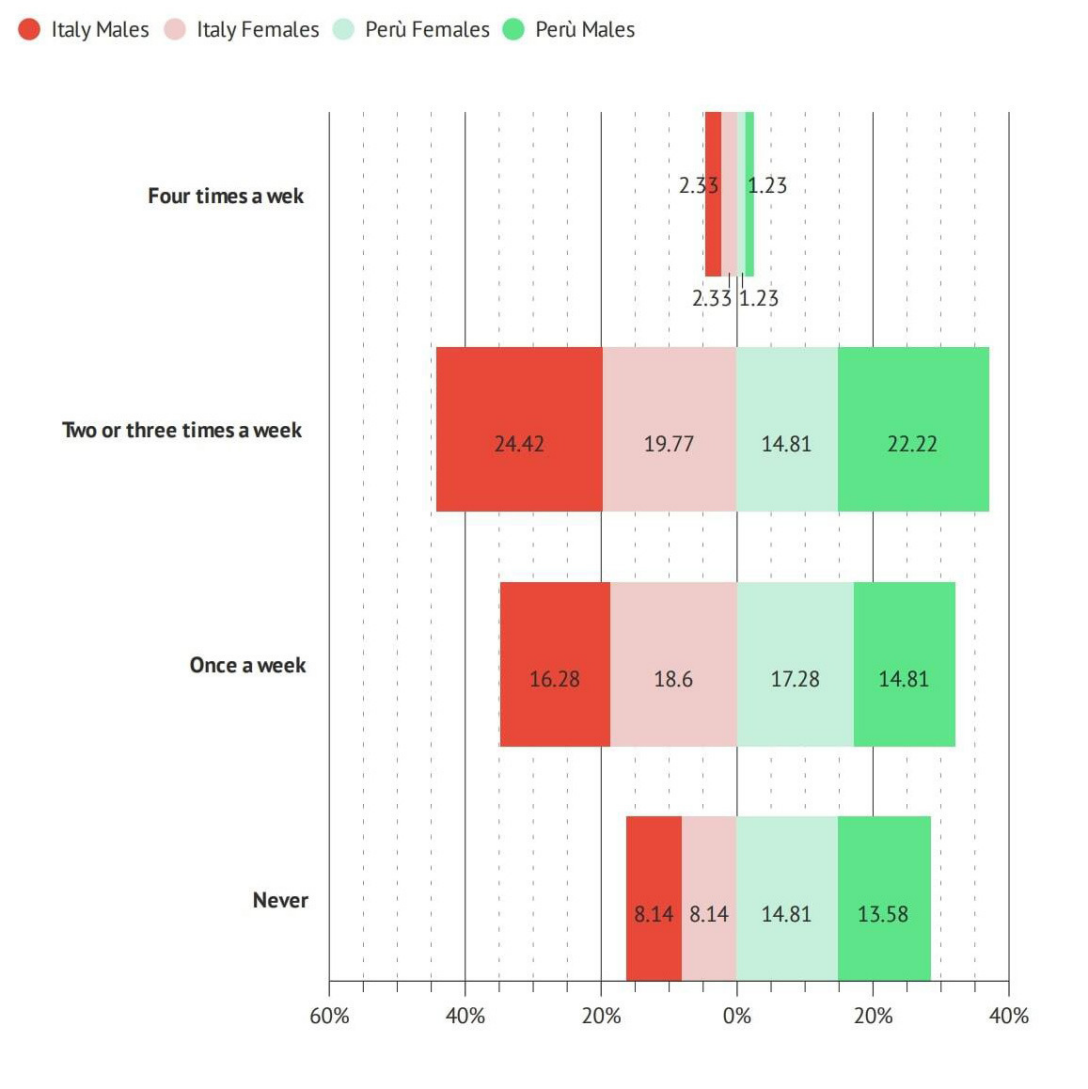
Percentage of dentistry professionals engaging in physical activity (times a week).

In detail, 44% of Italian vs. 37% of Peruvians respondents perform physical activity two/three times a week (Figure 5).

While no physical activity was performed by ~15.1% of surveyed operators in Italy and 22.2 % in Perù (*p* < 0.05).

### Musculoskeletal pain prevalence

**Figure 6:**
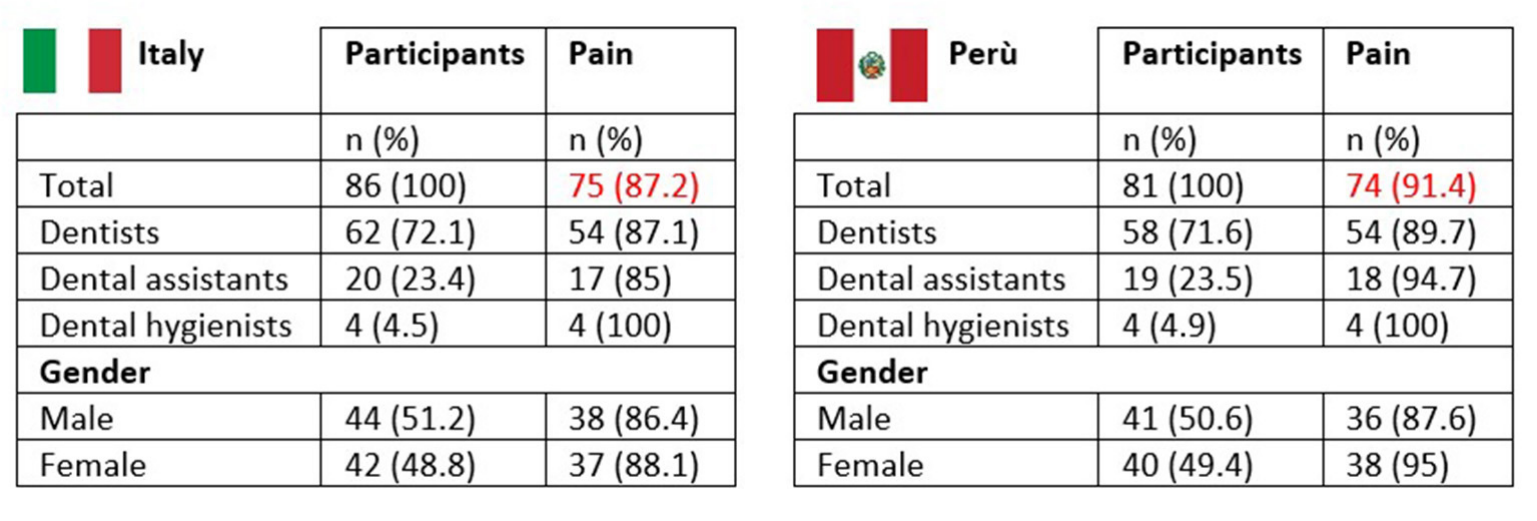
Percentage of dentists who complain about musculoskeletal pain.

The percentage of dentists who complain about musculoskeletal pain is 87.2% in Italy and 91.4% in Perú (*p* < 0.05). Respectively, 88.1% of females and 86.4% of males in Italy (ns), while 95% of females and 87.8% of males in Perú (*p* < 0.05) (Figure 6).

### Timing of pain onset

**Figure 7:**
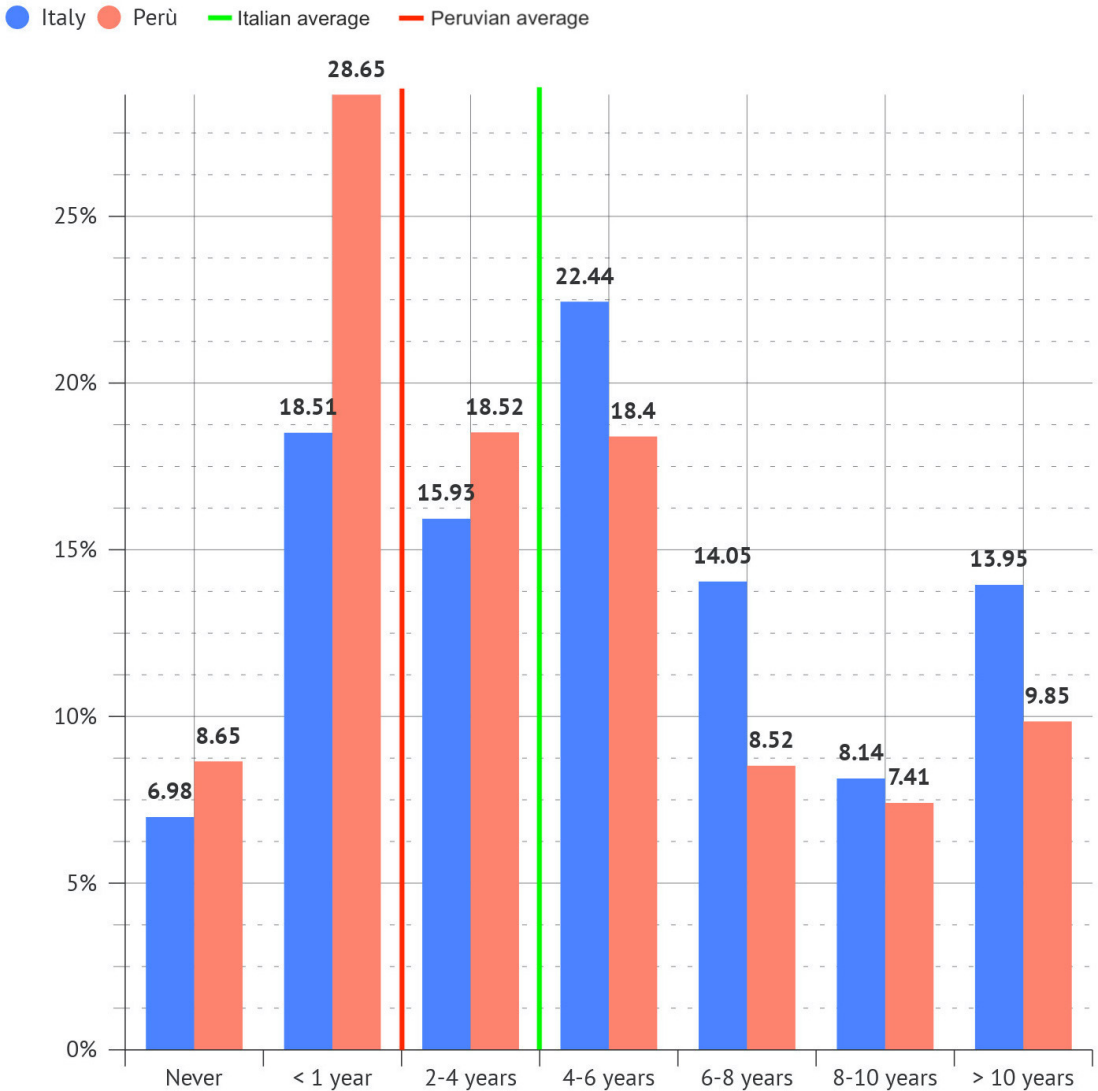
Period of dental practice after which musculoskeletal pain occurred.

The average musculoskeletal timing of pain onset, from the very beginning of work activity, in the two groups was, respectively, 3 years (±0.4) in Italy and 1.8 years (±0.4) in Perú (*p* < 0.05; Figure 7).

### Pain distribution according to working positions

The most frequent areas where musculoskeletal pain occurred were: the neck (~60%), lumbar region (52.1%), shoulder (43.3%), dorsal region (37.7%), and wrist (30.6%). Elbow, hip, knee, and ankle were less frequently involved.

Dental operators showed similar percentages of musculoskeletal pain in the cervical, dorsal and lumbar region, and in the shoulder, elbow, knee, and hip in Italy and Perú.

**Figure 8:**
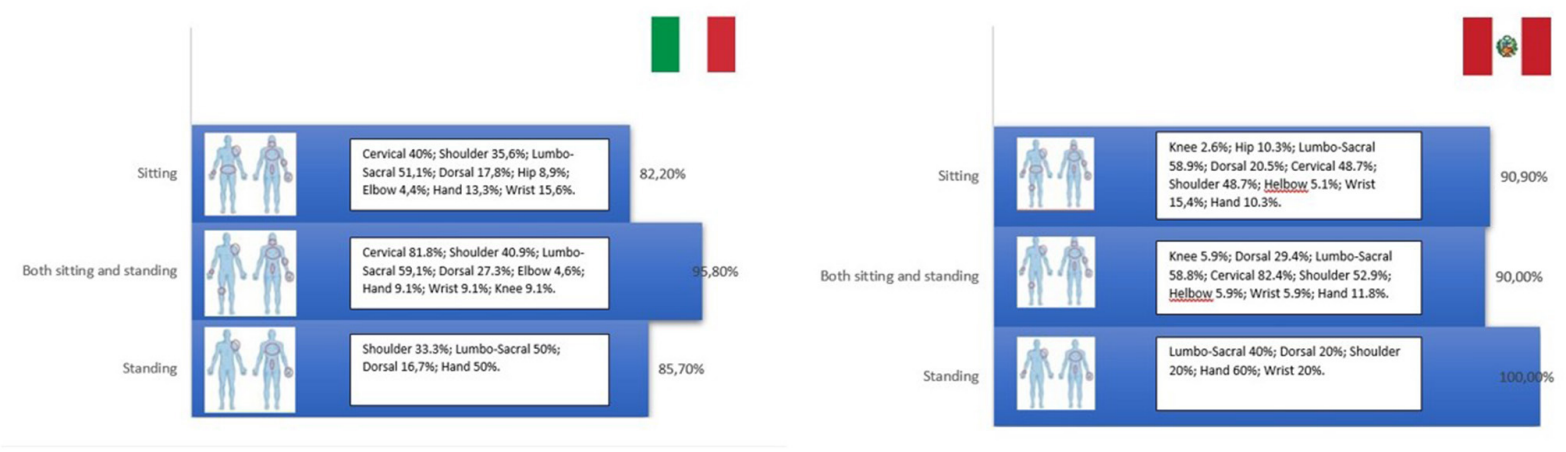
The most frequent areas where musculoskeletal pain occurred and relative percentages.

According to the working position (Figure 8), all workers who perform their activity in a seated position complain of pain 82.2% in Italy and 90.9% in Perú. Furthermore, pain is distributed mainly in the lumbar (51.1 % in Italy vs. 58.9% in Perú) and cervical region (40% in Italy vs. 48.7%) in Perú. All dentists in a stand-up position complain of pain in 85.7% of Italy and 100% of Perú. Maintaining a stand-up position, pain is located in the lumbar region (50% in Italy vs. 40% in Perú) and the hand (50% in Italy vs. 60% in Perú). Concerning dentists working half in a seated position and half in a stand-up position, the pain was detected mainly in the cervical (81.1% in Italy vs. 82.4% in Perú) and lumbar (59.1% in Italy vs. 58.8% in Perú) regions and in the shoulder (40.9% in Italy vs. 52.9% in Perú).

### Pain and working hours

Higher working hours per day and week demonstrated a more significant percentage of musculoskeletal pain in Italy and Perú. In particular, operators work more than 8 h daily and more than 40 h weekly. The years worked showed a predisposition to developing pain over time, with the highest percentages after 10–20 years of work (94.9%) in Italy and Perú. Females showed significantly higher rates concerning occupation, working hours per day and years of work (*p* < 0.05).

### Pain—Work inability

**Figure 9:**
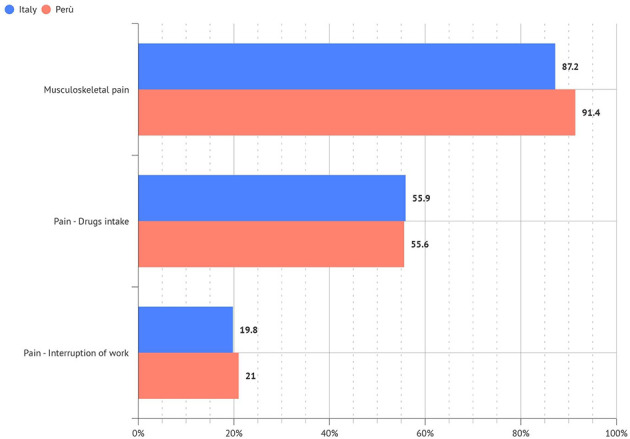
Relation between pain, working hours, work inability, and drug consumption.

19.8% of people in Italy and 10.5% in Perú complained of inability to work due to musculoskeletal pain (*p* < 0.05; Figure 9).

### Pain—Drug consumption

55.9% of people in Italy and 30% in Perú used medicine to keep working without pain (Figure 9).

## Discussion

This study represents the updated snapshot describing the prevalence of musculoskeletal pain affecting Italian and Peruvian dental professionals. Our paper lays the foundations on a theory-model of work-related diseases which clearly explains the relationship with forced posture movements of low-load, rapid rhythms and high repetition for a long duration, all leading to orthopedic disorders. We employed a validated survey to make inferences about possible relationships or gather preliminary data to support further research and experimentation concerning dentists. Secondly, the questionnaire in the form of a survey makes it possible to obtain something of paramount importance during the pandemic without direct contact with the recipient. Even if the methodology was not conducted in person and we do not employ a validated questionnaire because it doesn't exist, the reliability of the questionnaire was assured because the software maintains the respondent's geographical traceability by registering the IP and does not allow the questionnaire to be filled in more than once.

Since most respondents were specialized dentists and general practitioners and only a minority were dental assistants and hygienists, this study could be considered a partial picture of the Italian and Peruvian dentist operators. On the contrary, the dentist category was well represented in Italy, while in Perú, 35% could not find a precise operating area. This aspect could be considered a cultural factor affecting the differences between the two countries.

The principal result of our study highlights the prevalence of pain at the level of the musculoskeletal system in both Italian and Peruvian populations.

**Figure 10:**
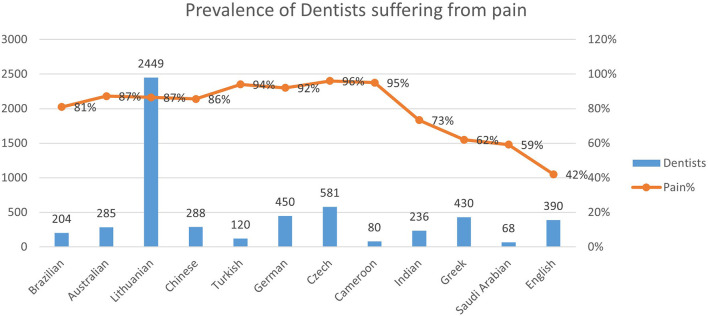
Previous studies showing the prevalence of dentists suffering from musculoskeletal pain.

Previous studies (Figure 10) have shown a prevalence of 81.4% in a population of 204 Brazilian dentists (13), 87.2% in 285 Australian dentists (3), 86.5% in 2,449 Lithuanian dentists (14) and 85.6% in 288 Chinese dentists (15). Other studies are reporting higher prevalence: 94% in 120 Turkish dentists (16), 92% in 450 German dentists and dental students (17), 96% in 581 Czech dentists (18) and 95% in 80 dentists of Cameroon (19). Only a few studies show a low prevalence: 73.3% in 236 Indian dentists (20), 62% in 430 Greek dentists (21), 59.2% in 68 Saudi Arabian dentists (22) and 42% in 390 students of dentistry in England (23).

Our study offers an updated panorama of two different realities that have never been considered before detecting musculoskeletal pain related to dental practice in 87.2% of Italian dentists and 91.4% of Peruvian ones. The percentage of dentists suffering from pain is comparable to those in other countries (3, 13–19). The data above are beneficial to help the scientific community (R1-4) both confirm the theory-model of work-related diseases and understand how the problem of disorders at the level of the musculoskeletal system must be addressed worldwide. A weak point of our study could be the small number of subjects questioned. However, it is necessary to consider how the pandemic has slowed down research worldwide.

Another interesting issue is that the onset of pain is more significant in Italy, and this data could be considered the consequence of more excellent know-how of ergonomics. This subject is explained during the academic path in Italy and is regarded as the main framework of prevention.

Our study confirms a high correlation between the nature of working conditions, which can negatively affect musculoskeletal pain development. A weak point of our study was that we needed to analyse working conditions over time and find a statistical correlation between pain onset and specific risk factors. Our survey was instrumental in obtaining necessary information about pain distribution according to working positions. The dentist's work often includes tools that force the subject to maintain an incorrect posture that overloads different anatomical sites (24). Our study mapped the anatomical region of pain in a seated position, stand-up position and half seated and half stand-up position, identifying some trends in the Italian and Peruvian groups.

In a seated position, a higher musculoskeletal pain prevalence was found in the lumbar (51.1 % in Italy vs. 58.9% in Perú) and cervical region (40% in Italy vs. 48.7%) in Perú.

Furthermore, in all dentists maintaining a stand-up position, pain is mainly in the lumbar region (50% in Italy vs. 40% in Perú) and the hand (50% in Italy vs. 60% in Perú).

Concerning dentists working half in a seated position and half in a stand-up position, the pain was detected mainly in the cervical (81.1 % in Italy vs. 82.4% in Perú) and lumbar (59.1% in Italy vs. 58.8% in Perú) regions and in the shoulder (40.9% in Italy vs. 52.9% in Perú).

In our study, the prevalence of cervical pain agrees with previous studies that show a higher prevalence of pain in the cervical region (17) but has a similar percentage of lumbosacral pain. Furthermore, our analysis considers the interplay between the working position and the prevalence of pain in a specific anatomical site for the first time.

The medical literature documents how the cervical tract is the most exposed to the development of musculoskeletal disorders related to dental activity. This finding is justified by the posture during work with the head tilted forward by 15/20 degrees, resulting in the muscular overload of the neck and the cervical spine joints (6). In this posture, the back muscles of the neck work with a biomechanical disadvantage in fulfilling their role in maintaining the head erect against gravity, causing contracture and pain in the neck (cervical tract). It is also noted how this posture, over time, determines changes in cervical lordosis. Loss of cervical lordosis can cause pain, functional disabilities and disc protrusion. This biomechanical issue is in line with our findings. A higher percentage of cervical pain was detected in seated positions and half-seated and half-stand-up positions in Italy and Perú.

At the level of the lumbar-sacral region, work-related disorders of the musculoskeletal system are mainly determined by the progressive reduction of the lordosis curve due to incorrect posture in sitting position, the lack of retroversion for anteriorisation during sitting associated with weakness of the stabilizing muscles always determined by prolonged sitting with incorrect posture. Our study found a high pain percentage in Italians and Peruvians. As shown by previous works, this anatomical district is involved in all categories of professionals equally and without any difference between males and females in Italy, but with a higher prevalence of females in Peru.

Musculoskeletal disorders related to dental activity were detected at the shoulder level with a prevalence of 40.9% in Italy vs. 52.9% in Perú, mainly in dentists working half seated and half in stand-up position.

Our data align with those found in previous studies (21, 22, 30, 31).

These shoulder disorders could be related to repetitive movements, vibrations during instruments, and the long duration of the procedures. The biomechanical substrate is identified in the forced and prolonged posture over time. The shoulder in suspension abducted with an angle of 45 degrees; the elbow flexed and pronated with the isometric and eccentric contraction of the deltoid, supraspinatus, trapezius and anterior serratus. Although the literature does not indicate effective preventive intervention in our work (25, 28–31), we have interpreted a lower prevalence in Italy due to the employment of modern instruments and greater know-how of ergonomics in maintaining correct posture.

In previous work, physical activity has been recognized as particularly effective in preventing musculoskeletal disorders (26). We cannot find a significant correlation with a specific sport in our work. Our work certainly has limitations related to the methodology that only allows us to deepen the clinical picture of the subjects and the type of study. This cross-sectional study presents only a snapshot that can describe the prevalence in that period well. Our study offers a reliable picture of the overall sedentary population among the two groups: 15.1% in Italy and 22.2% in Perú (*p* < 0.05). If inactive people worldwide are about 17% (27), we can consider the Peruvian dentist highly at risk.

Another important finding is that even if 82.7% of Italian subjects had pain during professional activity, only 19.8% stopped their activity, while 55.9% continued due to drug consumption (e.g. painkillers). The situation above is mirrored with Peruvian subjects, where 91.4% complained of pain during activity, only 10.5% suspended their activity, and 30% took drugs to continue working. This is an important fact that underlines how a category that works in the freelance profession and for which suspending the activity means reducing income prefers to continue enduring pain or taking a drug. Furthermore, this consideration has a greater weight in Peru than in Italy due to different economic scenarios not being considered in this paper.

## Conclusions

Thanks to the cross-sectional design, our study represents a necessary and faithful photograph that describes the prevalence of musculoskeletal disorders related to dentistry in Italy and Perú, noting the extent of the problem and its widespread diffusion worldwide. Moreover, precisely due to the peculiar methodological characteristics, the coexistence of possible associated factors in the population studied must be considered in planning prevention interventions in areas we have shown to be most affected: cervical spine, lumbar spine and shoulders. Finally, we can conclude that this research is unique in comparing two populations that are very similar despite the geographical distance. There is a strong need to educate the operator with awareness campaigns in ergonomics, rest, and specific exercises to prevent overload and orthopedic pathology. Future studies will be necessary to understand better that this problem must be considered seriously, but the type of specific intervention to reduce the onset of these disorders and their impact on the community.

## Data availability statement

The raw data supporting the conclusions of this article will be made available by the authors, without undue reservation.

## Ethics statement

The studies involving human participants were reviewed and approved by Ethics approval (number 23). The patients/participants provided their written informed consent to participate in this study.

## Author contributions

MM submitted the study protocol to the ethics committee. FF, NF, FP, and MM selected the sample. RS analyzed data. MM, NF, and FP wrote the manuscript. All authors read and approved the final manuscript.

## Supplementary material

Supplementary Table 1 18-question multiple-choice questionnaire administered to Italian and Peruvian participants.

## References

[1] Lee CY Wu JH Du JK. Work stress and occupational burnout among dental staff in a medical centre. J Dent Sci. (2019) 14:295–301. 10.1016/j.jds.2019.01.006 31528258 PMC6739458

[2] Moodley R Naidoo S Wyk JV. The prevalence of occupational health-related problems in dentistry: a review of the literature. J Occup Health. (2018) 60:111–25. 10.1539/joh.17-0188-RA 29213011 PMC5886878

[3] Leggat PA Smith DR. Musculoskeletal disorders self-reported by dentists in Queensland, Australia. Aust Dent J. (2006) 51:324–7. 10.1111/j.1834-7819.2006.tb00451.x 17256307

[4] Leggat PA Kedjarune U Smith DR. Occupational health problems in modern dentistry: a review. Ind Health. (2007) 45:611–21. 10.2486/indhealth.45.611 18057804

[5] Identification and control of work-released diseases. Report of a WHO expert committee. World Health Organ Tech Rep Ser. (1985) 714:1–71. 3922127

[6] Luttmann A Jäger I Griefahn B Caffier G Liebers F Steinberg F. Preventing Musculoskeletal Disorders in the Workplace; Protecting Workers' Health Series, 5. Geneva: World Health Organization (2003).

[7] Gupta A Ankola AV Hebbal M. Dental ergonomics to combat musculoskeletal disorders: a review. Int J Occup Saf Ergon. (2013) 19:561–71. 10.1080/10803548.2013.11077005 24321635

[8] Chopra A. Musculoskeletal disorders in dentistry—a review. JSM Dent. (2014) 2:1032. 10.5958/j.2320-5962.2.1.016

[9] Kalluri A Puranik MP Uma SR. Musculoskeletal disorders in dental workplace: a comprehensive review. Int J Appl Dent Sci. (2018) 4:140–5.

[10] Hayes MJ Smith DR Cockrell D. An international review of musculoskeletal disorders in the dental hygiene profession. Int Dent J. (2010) 60:343–52. 21141207

[11] Lietz J Kozak A Nienhaus A. Prevalence and occupational risk factors of musculoskeletal diseases and pain among dental professionals in Western countries: a systematic literature review and meta-analysis. PLoS ONE. (2018) 13:0208628. 10.1371/journal.pone.0208628 30562387 PMC6298693

[12] De Sio S Traversini V Rinaldo F Colasanti V Buomprisco G Perri R. Ergonomic risk and preventive measures of musculoskeletal disorders in the dentistry environment: an umbrella review. PeerJ. (2018) 6:4154. 10.7717/peerj.4154 29362689 PMC5772380

[13] Ísper Garbin AJ Barreto Soares G Moreira Arcieri R Adas Saliba Garbin C Siqueira CE. Musculoskeletal disorders and perception of working conditions: a survey of Brazilian dentists in São Paulo. Int J Occup Med Environ Health. (2017) 30:367–77. 10.13075/ijomeh.1896.00724 28481371

[14] Puriene A Aleksejuniene J Petrauskiene J Balciuniene I Janulyte V. Self-reported occupational health issues among Lithuanian dentists. Ind Health. (2008) 46:369–74. 10.2486/indhealth.46.369 18716385

[15] Yi J Hu X Yan B Zheng W Li Y Zhao Z. High and specialty-related musculoskeletal disorders afflict dental professionals even since early training years. J Appl Oral Sci. (2013) 21:376–82. 10.1590/1678-775720130165 24037079 PMC3881886

[16] Polat Z Bas kan S Altun S Tacir I. Musculoskeletal symptoms of dentists from South-East Turkey. Biotechnol Biotechnol Equip. (2007) 21:86–90. 10.1080/13102818.2007.10817421

[17] Ohlendorf D Naser A Haas Y Haenel J Fraeulin L Holzgreve F. Prevalence of musculoskeletal disorders among dentists and dental students in Germany. Int J Environ Res Public Health. (2020) 17:8740. 10.3390/ijerph17238740 33255491 PMC7727829

[18] Sustová Z Hodacová L Kapitán M. The prevalence of musculoskeletal disorders among dentists in the Czech Republic. Acta Med. (2013) 56:150–6. 10.14712/18059694.2014.10 24693796

[19] Agbor AM Hilbert K. Work-related musculoskeletal disorders amongst oral health workers in Cameroon. OHDM. (2016) 15:1–6.

[20] Atri M Nagraj A. Identifying musculoskeletal disorders amongst dentists—the need for the hour. Int J Med Sci Public Health. (2014) 6:730–4. 10.5455/ijmsph.2014.010420144

[21] Alexopoulos EC Stathi IC Charizani F. Prevalence of musculoskeletal disorders in dentists. BMC Musculoskelet Disord. (2004) 5:16–23. 10.1186/1471-2474-5-16 15189564 PMC441388

[22] Abduljabbar TA. Musculoskeletal disorders among dentists in Saudi Arabia. Pakistan Oral Den J. (2000) 28:135–44.31308760

[23] Vijay S Ide M. Musculoskeletal neck and back pain in undergraduate dental students at a UK dental school—a cross-sectional study. Br Dent J. (2016) 221:241–5. 10.1038/sj.bdj.2016.642 27608577

[24] Bozkurt S Demirsoy N Günendi Z. Risk factors associated with work-related musculoskeletal disorders in dentistry. Clin Investig Med. (2016) 39:27527. 10.25011/cim.v39i6.27527 27917817

[25] Mulimani P Hoe VC Hayes MJ Idiculla JJ Abas AB Karanth L. Ergonomic interventions for preventing musculoskeletal disorders in dental care practitioners. Cochrane Database Syst Rev. (2018) 10:CD011261. 10.1002/14651858.CD011261.pub2 30320459 PMC6516890

[26] Letafatkar A Rabiei P Alamooti G Bertozzi L Farivar N Afshari M. Effect of therapeutic exercise routine on pain, disability, posture, and health status in dentists with chronic neck pain: a randomized controlled trial. Int Arch Occup Environ Health. (2020) 93:281–90. 10.1007/s00420-019-01480-x 31654125

[27] Kohl HW 3rd Craig CL Lambert EV Inoue S Alkandari JR Leetongin G. The pandemic of physical inactivity: global action for public health. Lancet. (2012) 380:294–305. 10.1016/S0140-6736(12)60898-8 22818941

[28] Jain R Rana KB Meena ML Verma V. Application of the best-worst method approach for prioritizing risk factors of musculoskeletal disorders among mobile device users: a case study. Work. (2022) 73:559–568 10.3233/WOR-205148 35938262

[29] Jain R Meena ML Rana KB. Risk factors of musculoskeletal symptoms among mobile device users during work from home. Int J Occup Saf Ergon. (2021) 28:2262–8. 10.1080/10803548.2021.1979318 34514964

[30] Macrì M Murmura G Scarano A Festa F. Prevalence of temporomandibular disorders and its association with malocclusion in children: a transversal study. Front Public Health. (2022) 10:86083. 10.3389/fpubh.2022.860833 36159244 PMC9500209

[31] Festa F Rotelli C Scarano A Navarra R Caulo M Macrì M. Functional magnetic resonance connectivity in patients with temporomadibular joint disorders. Front Neurol. (2021) 12:629211. 10.3389/fneur.2021.629211 33912123 PMC8072218

