# Supplementary material for: Interpreting the prevalence of musculoskeletal pain impacting Italian and Peruvian dentists likewise: A cross-sectional study
Source: Front Public Health. 2023 Feb 9;11:1090683. doi: 10.3389/fpubh.2023.1090683 (PMC9947776; doi:10.3389/fpubh.2023.1090683)
Supplement: Supplementary Table 1 — 18-question multiple-choice questionnaire administered to Italian and Peruvian participants. [file Data_Sheet_1.PDF]

MUSCULOSKELETAL PAIN DURING DENTAL PRACTICES

1. *Are you male or female?*
   - Male
   - Female
2. *How old are you?*
3. *Profession*
   - Dentist
   - Dental assistant
   - Dental hygienist
4. *If you are a dentist, what is your operative area?*
   - Dentist surgeon
   - Dentist specialised in prosthetics
   - Dentist specialised in odontopediatrics
   - Orthodontist
   - General Dentist
5. *How long do you practice the profession?*
   - <1 year
   - 2 years
   - 4 years
   - 6 years
   - 8 years
   - 10 years
   - >10 years
   - Other (specify)
6. *How many hours a day do you work?*
   - <4 hours
   - 4-6 hours
   - 6-8 hours
   - 8-10 hours
   - 10-12 hours
   - >12
   - Other (specify)
7. *What is the dominant limb you work with?*
   - Right-handed
   - Left-handed
   - Ambidextrous
8. *How do you do your job? (Bar more options)*
   - Without assistant
   - With assistant
   - With direct method: without the mirror
   - With indirect method: with the mirror
   - With the mirror sometimes
   - Standing
   - Sitting
   - Half-time standing half time sitting
   - Work with a magnifying glass
   - Work without a magnifying glass
9. *Do you do physical activity?*
   - Never
   - Once a week
   - 2-3 times a week
   - 4 times a week
   - More than 4 times a week
10. *How long do you do the physical activity each time?*
    - <30 minutes
    - 30-60 minutes
    - >60 minutes
11. *What kind of physical activity do you do?*
    - Free body gymnastics/stretching
    - Gym/weight
    - Swim
    - Martial arts
    - Tennis
    - Golf
    - Gymnastics at home with internet
    - Race/jogging
    - Gardening
    - Ski
    - Bike
    - Dance
12. *Do you ever had musculoskeletal pain?*
    - No
    - Yes
    - I do not know
13. *How long after the start of your activity did the musculoskeletal pain develop?*
    - <1 year
    - 4-6 years
    - 6-8 years
    - >10 years
    - Other (Specify)
14. *Do you have work-related musculoskeletal pain?*
    - No
    - Before work
    - During work
    - After work
    - Ever
15. *Where do you feel pain? In which anatomical district?*
    - Feet
    - Ankle
    - Knee
    - Hip
    - Lumbosacral
    - Dorsal
    - Cervical
    - Shoulder
    - Elbow
    - Wrist
    - But no
    - Bilateral pain
16. *What therapies have you performed in the past?*
    - I have not performed therapies
    - Laser Therapy
    - Infiltrations of Hyaluronic Acid
    - Tecar Therapy
    - Physiotherapy with a Physiotherapist
    - Gym activities with Trainer / Science
    - Motor
    - Regenerative Medicine
    - Other (specify)
17. *Have you ever had to stop work for musculoskeletal pain?*
    - Yes
    - No
18. *Have you ever taken medications to work without musculoskeletal pain?*
    - Yes
    - No
